# Association Between Positive Results on the Primary Care–Posttraumatic Stress Disorder Screen and Suicide Mortality Among US Veterans

**DOI:** 10.1001/jamanetworkopen.2020.15707

**Published:** 2020-09-03

**Authors:** Samantha A. Cooper, Benjamin R. Szymanski, Kipling M. Bohnert, Rebecca K. Sripada, John F. McCarthy

**Affiliations:** 1 Serious Mental Illness Treatment Resource and Evaluation Center, Office of Mental Health and Suicide Prevention, Department of Veterans Affairs, Ann Arbor, Michigan; 2 Department of Veterans Affairs Center for Clinical Management Research, VA Ann Arbor Healthcare System, Ann Arbor, Michigan; 3 Department of Psychiatry, University of Michigan, Ann Arbor

## Abstract

**Importance:**

Suicide rates are higher among veterans compared with nonveterans, and the prevalence of posttraumatic stress disorder (PTSD) is higher among veterans compared with the general adult population in the US. To date, no study has examined the association between PTSD screening results and suicide mortality among veterans.

**Objective:**

To examine whether veterans receiving care in the US Veterans Health Administration (VHA) health system who had positive results on the Primary Care–Posttraumatic Stress Disorder Screen (PC-PTSD) had a greater risk of suicide mortality compared with those who had negative results and to assess whether such risk decreased over time.

**Design, Setting, and Participants:**

Multivariable proportional hazards regression models were used to evaluate suicide mortality risk through December 31, 2016, among a cohort of veterans who received the PC-PTSD in the VHA health system. The VHA administers the PC-PTSD to patients nationwide, and screening results are routinely documented in the VHA Corporate Data Warehouse. The PC-PTSD includes 4 questions regarding PTSD symptoms, to which patients respond with either a positive (yes) or negative (no) answer. All patients who completed the PC-PTSD in 2014 and who did not have a diagnosis of PTSD in the year before screening were included in the analysis. A score of 3 or 4 on the PC-PTSD indicated a positive result, and a score of 0, 1, or 2 indicated a negative result. Data collection and analyses were performed from November 13, 2018, to June 18, 2019.

**Exposures:**

Primary Care–Posttraumatic Stress Disorder Screen (PC-PTSD).

**Main Outcomes and Measures:**

Suicide mortality risk, as assessed through data obtained from the US Veterans Affairs/Department of Defense Mortality Data Repository.

**Results:**

A total of 1 693 449 PC-PTSDs were completed by 1 552 581 individual veteran patients in 2014. Most of the patients were White (73.9%), married (52.2%), male (91.1%), 55 years or older (62.5%), and had completed only 1 PC-PTSD (92.1%). In multivariable analyses, positive PC-PTSD results (ie, total scores of 3 or 4) were associated with a 58% increase in the risk of suicide mortality at 1 day after screening (hazard ratio [HR], 1.58; 95% CI, 1.19-2.10) and a 26% increase in the risk of suicide mortality at 1 year after screening (HR, 1.26; 95% CI, 1.07-1.48). A positive response on item 4 (“felt numb or detached from others, activities, or your surroundings”) of the PC-PTSD was associated with a 70% increase in suicide mortality risk at 1 day after screening (HR, 1.70; 95% CI, 1.27-2.28).

**Conclusions and Relevance:**

Positive PC-PTSD results, and specifically reports of feeling numb or detached, were associated with increases in the risk of suicide mortality. These associations decreased over time. The findings of this study can inform interpretation of PC-PTSD responses and suggest the importance of recent improvements made to the VHA suicide risk assessment.

## Key Points

**Question:**

Are positive results on the Primary Care–Posttraumatic Stress Disorder Screen associated with an increase in the risk of suicide mortality among veterans receiving care in the US Veterans Health Administration system and, if so, does this risk decrease over time?

**Findings:**

In this cohort study of 1 552 581 veteran patients receiving care in the US Veterans Health Administration system who were followed up through 2016, analyses of 1 693 449 screens for posttraumatic stress disorder administered in 2014 indicated a statistically significant 58% increase in the risk of suicide mortality at 1 day after a positive screening result. This risk of suicide mortality decreased over time.

**Meaning:**

The study's findings indicate that additional mental health and suicide risk assessment may be warranted after a patient receives a positive result on the Primary Care–Posttraumatic Stress Disorder Screen.

## Teaser

This cohort study uses data from the Mortality Data Repository to examine the association between positive results on the Primary Care–Posttraumatic Stress Disorder Screen and the risk of suicide mortality among veterans receiving care in the US Veterans Administration health system.

## Introduction

Posttraumatic stress disorder (PTSD) is one of the signature wounds of war among veterans who served in the conflicts in Afghanistan and Iraq. The estimated lifetime prevalence of PTSD among veterans is 11% to 12%,^1^ nearly double that of the general adult population in the US, which is 6% to 8%.^2,3,4^ Posttraumatic stress disorder is associated with an increased risk of morbidity^5,6,7^ and mortality.^8^ Among veterans, PTSD is associated with increased impairment in psychosocial functioning, decreased health-associated quality of life,^9^ and increased mortality.^10,11,12,13,14,15,16^

Veterans also have an increased risk of suicide.^17^ Suicide prevention is the first clinical priority of the US Department of Veterans Affairs. Posttraumatic stress disorder is associated with increases in suicidal ideation and suicide attempts.^18,19^ Findings regarding the association between PTSD and suicide mortality among veterans have differed across studies, in part depending on the covariates included in the analyses. In general, analyses without adjustments for mental health comorbidities have reported positive associations between a diagnosis of PTSD and suicide risk,^12,13,14,16^ while analyses with adjustments for mental health comorbidities have observed negative associations.^16,20,21,22,23^

Previous studies have typically relied on PTSD diagnoses reported in medical records; however, patients may experience clinically important PTSD symptoms without receiving a diagnosis,^24^ and PTSD symptoms may fluctuate over time.^25^ Therefore, it may be informative to evaluate the suicide mortality risk associated with self-reported PTSD symptoms among veterans without an established PTSD diagnosis. Recent studies have identified positive associations between PTSD symptoms and both suicidal ideation and suicidal behaviors^26,27,28,29,30,31,32^; however, to our knowledge, no study has examined whether veteran-reported PTSD symptoms are associated with the risk of suicide mortality.

The US Veterans Affairs/Department of Defense Clinical Practice Guidelines recommend periodic PTSD screening, including annual assessments during the first 5 years after military separation and every 5 years thereafter. The US Veterans Health Administration (VHA) primarily uses the Primary Care–Posttraumatic Stress Disorder Screen (PC-PTSD) for its annual PTSD screening. This screen was based on criteria from the *Diagnostic and Statistical Manual of Mental Disorders* (Fourth Edition) and was developed to quickly identify patients with PTSD who receive primary care in the VHA heath system.^33^ The PC-PTSD includes only 4 items, facilitating its administration during routine care, which most often occurs in primary care settings.

To our knowledge, no study has examined the association between PTSD symptoms and suicide mortality. Information regarding suicide mortality risk after the administration of the VHA PC-PTSD may inform veteran suicide prevention activities and provide new information regarding the association between PTSD symptoms and suicide. We hypothesized that positive PC-PTSD results (ie, total scores of 3 or 4) would be associated with suicide mortality. Because PTSD symptoms may fluctuate over time, we further hypothesized that associations between reports of symptoms and suicide mortality risk would decrease over time.

## Methods

Using the VHA Corporate Data Warehouse, we identified 1 837 114 completed PC-PTSD screens in 2014. Data regarding vital status and cause of death were obtained from the joint US Veterans Affairs/Department of Defense Mortality Data Repository. Screens were excluded if data on vital status and cause of death were unavailable or if the recorded date of death was before or on the date of the PC-PTSD. Additional exclusion criteria included having no record of a VHA inpatient or outpatient encounter on the screening date, administration of the PC-PTSD at a facility outside of the 50 US states and the District of Columbia, documented death in Puerto Rico or the Virgin Islands, missing or invalid data on patient age (ie, ages <18 years or >115 years), and nonveteran status as indicated by VHA patient records and Department of Defense personnel records. The analyses were conducted as part of ongoing VHA operations and program evaluation and were approved by the Veterans Affairs Office of Mental Health and Suicide Prevention with a waiver of informed consent. This report followed the Strengthening the Reporting of Observational Studies in Epidemiology (STROBE) reporting guideline for cohort studies.

A total of 4 items are included in the PC-PTSD. Each item is a response to the question, “In your life, have you ever had any experience that was so frightening, horrible, or upsetting that”: (1) “in the past month, you have had nightmares about it or thought about it when you did not want to?”; (2) “tried hard not to think about it or went out of your way to avoid situations that reminded you of it?”; (3) “were constantly on guard, watchful, or easily startled?”; and (4) “felt numb or detached from others, activities, or your surroundings?” Patients respond to each item with an answer of yes (considered a positive response) or no (considered a negative response). The PC-PTSD has been reported to have high diagnostic validity in primary care and mental health care settings.^33,34,35^

For the main analysis, we limited the cohort of screens to those administered to veterans without a PTSD diagnosis (*International Classification of Diseases, Ninth Revision, Clinical Modification* [*ICD-9-CM*] code 309.81) associated with a VHA inpatient stay or outpatient encounter in the 365 days before each PC-PTSD was administered. These criteria identified 1 693 449 total screens administered to 1 552 581 individual patients.

Suicide mortality was measured from 1 day after each PC-PTSD administration date through December 31, 2016. Data on patient mortality and cause of death were obtained from the Mortality Data Repository, and suicide death was defined by *International Statistical Classification of Diseases and Related Health Problems, Tenth Revision* codes U03, X60 to X84, and Y87.0.

Most covariate data were collected from the Corporate Data Warehouse and the VHA National Patient Care Database. Patient age and sex were obtained from the Mortality Data Repository and, if missing, from medical records. Data on suicide attempts were collected from the Veterans Affairs Suicide Prevention Applications Network^36^ database as well as medical records. At the patient level, risk factor data collected were age, sex, service-connected disability status for PTSD, marital status, race, and mental health diagnoses. Age was assessed on January 1, 2014. The service-connected disability compensation program provides tax-free monthly benefits to veterans for disabilities that were incurred or aggravated during military service.^37^ Service-connected disability status for PTSD was defined as an effective date of disability status that occurred before the date of the patient’s initial PC-PTSD in 2014. The presence of other mental health diagnoses was assessed in the 365 days before and including the initial PC-PTSD administration date in 2014. Specific *ICD-9-CM* codes for identifying the presence of mental health diagnoses are available in eTable 1 in the Supplement.

At the screening level, 6 indicator variables were created to categorize the types of care patients received on each PC-PTSD administration date: primary care services through the Primary Care–Mental Health Integration program, primary care services outside of the Primary Care–Mental Health Integration program, specialty mental health care services, other outpatient services, inpatient mental health stay, and other inpatient stay. Variables were created to capture emergency department or inpatient mental health stays as well as the number of emergency department visit days and inpatient mental health bed-days in the 365 days before the screening date. An indicator variable was created for the presence of a documented suicide attempt in the 365 days before and including the screening date when the patient had an *ICD-9-CM* diagnosis code of E95.0-E95.8 in the medical records or when a suicide attempt was documented in the Suicide Prevention Applications Network database. Indicator variables were also created to record prescription fills for antipsychotic, antidepressant, benzodiazepine, mood stabilizer, and anxiolytic or sedative medications in the 365 days before and including the screening date.

Responses for each PC-PTSD were prepared for analysis in 3 ways. First, an indicator variable was created to record whether each PC-PTSD had a positive (total score of 3 or 4)^33^ or negative (total score of 0, 1, or 2) result. Second, each of the 4 items was treated as an individual indicator based on whether that item received a positive or negative response. Third, each screen was assigned a score (ranging from 0-4) for the total number of items with positive responses.

### Statistical Analysis

For descriptive analyses, frequencies were calculated, both overall and by suicide mortality status, for all variables among 1 552 581 patients. In bivariate analyses, the responses on the patient’s initial PC-PTSD in 2014 were examined by suicide mortality status through December 31, 2016. Fisher exact tests and χ^2^ tests were used to assess the differences in proportions, and Kruskal-Wallis tests were used to evaluate differences in means. Suicide rates per 100 000 person-years were calculated at the patient level by multiplying the number of observed suicides by 100 000, then dividing that value by the number of person-years at risk from the day after each patient’s initial PC-PTSD in 2014 until death or December 31, 2016, whichever occurred first.

In the proportional hazards regression analyses, each PC-PTSD screen was used as the unit of analysis. The patient’s time at risk began the day after the PC-PTSD was administered and ended at death or on December 31, 2016, whichever occurred first. We used partially conditional proportional hazards regression models^38^ and controlled for nonsuicide mortality as a competing risk. By treating each screen as the unit of analysis, the models were conditioned on the baseline covariates (age, sex, PTSD service connection, marital status, race, and mental health diagnoses) but not on the time-varying covariates (PC-PTSD responses, types of care received, and presence of a previous suicide attempt).

Five main proportional hazards regression models were used to examine suicide mortality risk by comparing positive (total score of 3 or 4) vs negative (total score of 0-2) PC-PTSD results, with adjustment for varying levels of covariates. An additional set of models were used, in which each item on the PC-PTSD was included as the main effect. Because we hypothesized that the suicide mortality risk among those with positive PC-PTSD results would be higher during the time closer to the screening date, we included an interaction term between the main effect and time in all models. Covariates were included in the models to adjust for potential confounding variables in the association between PC-PTSD responses and suicide mortality.^39,40^ We adjusted for covariates using a stepped process to better understand the associations between the covariates, the focal indicator, and the outcome.

Covariance sandwich estimators were used to adjust for the nested nature of the data, with screens nested within individuals and screens of individual patients nested within VHA facilities. The proportional hazards assumption was evaluated for all models. A supplemental post hoc trend analysis was performed in which PC-PTSD scores were treated as a continuous variable.

Three sensitivity analyses were conducted for both sets of models. First, to examine how screening results were associated with the risk of suicide mortality regardless of a previous diagnosis of PTSD, we analyzed all models by using a larger cohort of 1 790 355 patients, in which we retained screens that were associated with a PTSD diagnoses in the previous 365 days. Second, to eliminate overlapping time at risk, we analyzed all models by censoring time at risk at each patient’s subsequent PC-PTSD administration. Third, to assess whether the elimination of repeated PC-PTSD administrations would alter the observed associations, we restricted the analyses to 1 552 581 initial PC-PTSD screens in 2014. None of the sensitivity analyses produced results that substantially differed from those observed in the main analysis. All tests were 2-sided and unpaired, with a significance threshold of α = .05. Data collection and analyses were conducted from November 13, 2018, to June 18, 2019. Data analyses were performed using SAS software, version 9.4 and the SAS Enterprise Guide, version 7.1 (SAS Institute Inc).

## Results

Among 1 552 581 patients included in the analysis, 73.9% were White, 52.2% were married, 91.1% were male, and 62.5% were 55 years or older (Table 1). Most patients (92.1%) in the cohort received only 1 PC-PTSD in 2014, and the maximum number of screens received per person was 23. The number of days at risk ranged from 1 to 1095 (ie, 3 years), with a median time at risk of 904 days (interquartile range, 809-997 days) for all screens. A total of 1423 patients (0.1%) died by suicide within the follow-up period. The unadjusted suicide mortality rate was 37.6 deaths per 100 000 person-years.

**Table 1.** Characteristics of Patients by Suicide Mortality

Characteristic	Patients, No. (%)	Died by suicide, No. (%)			Suicide mortality rate per 100 000 person-years
		Yes	No	*P* value^a^	
Total	1 552 581 (100)	1423 (0.1)	1 551 158 (99.9)	NA	37.6
Sex					
Male	1 414 950 (91.1)	1370 (96.3)	1 413 580 (91.1)	<.001	39.9
Female	137 631 (8.9)	53 (3.7)	137 578 (8.9)		15.4
Age, median (IQR), y	61 (45-70)	59 (42-71)	61 (45-70)	.03^b^	NA
Age group, y					
18-34	229 705 (14.8)	259 (18.2)	229 446 (14.8)	<.001	45.0
35-54	351 880 (22.7)	333 (23.4)	351 547 (22.7)		37.9
55-74	697 463 (44.9)	530 (37.3)	696 933 (44.9)		31.0
≥75	273 533 (17.6)	301 (21.2)	273 232 (17.6)		48.7
Marital status					
Divorced	324 879 (20.9)	391 (27.5)	324 488 (20.9)	<.001	49.2
Married	810 829 (52.2)	572 (40.2)	810 257 (52.2)		29.0
Never married	220 085 (14.2)	270 (19.0)	219 815 (14.2)		49.5
Separated	48 198 (3.1)	54 (3.8)	48 144 (3.1)		45.6
Widowed	87 074 (5.6)	94 (6.6)	86 980 (5.6)		47.1
Unknown	61 516 (4.0)	42 (3.0)	61 474 (4.0)		28.4
Race					
American Indian or Alaska Native	10 460 (0.7)	10 (0.7)	10 450 (0.7)	<.001	38.9
Asian	26 368 (1.7)	9 (0.6)	26 359 (1.7)		13.8
Black or African American	245 324 (15.8)	73 (5.1)	245 251 (15.8)		12.1
White	1 147 914 (73.9)	1179 (82.9)	1 146 735 (73.9)		42.2
≥2 races	83 853 (5.4)	114 (8.0)	83 739 (5.4)		57.9
Unknown	38 662 (2.5)	38 (2.7)	38 624 (2.5)		39.4
Service-connected disability status for PTSD	89 631 (5.8)	75 (5.3)	89 556 (5.8)	.42	34.1
Mental health diagnosis					
Substance use disorder	135 664 (8.7)	279 (19.6)	135 385 (8.7)	<.001	84.2
Anxiety	149 273 (9.6)	267 (18.8)	149 006 (9.6)	<.001	72.7
Bipolar disorder	30 818 (2.0)	107 (7.5)	30 711 (2.0)	<.001	140.8
Depression	265 675 (17.1)	453 (31.8)	265 222 (17.1)	<.001	69.8
Schizophrenia	19 136 (1.2)	44 (3.1)	19 092 (1.2)	<.001	94.1
Other psychiatric condition	102 165 (6.6)	69 (4.9)	102 096 (6.6)	.008	27.5
Characteristics at initial PC-PTSD in 2014					
Item 1: reexperiencing	211 336 (13.6)	248 (17.4)	211 088 (13.6)	<.001	47.6
Item 2: avoidance	200 035 (12.9)	249 (17.5)	199 786 (12.9)	<.001	50.4
Item 3: hyperarousal	256 574 (16.5)	297 (20.9)	256 277 (16.5)	<.001	46.8
Item 4: numbing	193 720 (12.5)	264 (18.6)	193 456 (12.5)	<.001	55.2
Positive PC-PTSD result	166 685 (10.7)	218 (15.3)	166 467 (10.7)	<.001	52.9
PC-PTSD score					
0	1 204 171 (77.6)	1021 (71.8)	1 203 150 (77.6)	<.001	34.9
1	107 137 (6.9)	102 (7.2)	107 035 (6.9)		38.8
2	74 588 (4.8)	82 (5.8)	74 506 (4.8)		44.6
3	61 388 (4.0)	80 (5.6)	61 308 (4.0)		52.7
4	105 297 (6.8)	138 (9.7)	105 159 (6.8)		53.0
Median (IQR)	0	0 (0-1)	0	<.001^b^	NA
Health care system encounter on screening date					
PC-MHI	39 232 (2.5)	65 (4.6)	39 167 (2.5)	<.001	66.7
Non–PC-MHI primary care	1 416 585 (91.2)	1191 (83.7)	1 415 394 (91.3)	<.001	34.5
Specialty mental health	110 259 (7.1)	237 (16.7)	110 022 (7.1)	<.001	86.7
Other outpatient service	801 791 (51.6)	697 (49.0)	801 094 (51.6)	.04	35.7
Inpatient mental health	6384 (0.4)	33 (2.3)	6351 (0.4)	<.001	208.8
Other inpatient service	8250 (0.5)	11 (0.8)	8239 (0.5)	.21	60.2
Inpatient mental health stay in previous 365 d	14 645 (0.9)	59 (4.2)	14 586 (0.9)	<.001	163.5
Bed-days, median (IQR)	0	0	0	<.001^b^	NA
Emergency department visit in previous 365 d	231 513 (14.9)	291 (20.5)	231 222 (14.9)	<.001	51.8
Median (IQR)	0	0	0	<.001^b^	NA
Type of medication prescription filled in previous 365 d					
Antipsychotic	60 397 (3.9)	154 (10.8)	60 243 (3.9)	<.001	106.5
Antidepressant	257 741 (16.6)	412 (29.0)	257 329 (16.6)	<.001	65.6
Benzodiazepine	94 035 (6.1)	208 (14.6)	93 827 (6.1)	<.001	92.0
Mood stabilizer	159 771 (10.3)	267 (18.8)	159 504 (10.3)	<.001	69.0
Anxiolytic or sedative	136 235 (8.8)	276 (19.4)	135 959 (8.8)	<.001	83.8
Previous suicide attempt	3346 (0.2)	34 (2.4)	3312 (0.2)	<.001	412.1

Abbreviations: IQR, interquartile range; NA, not applicable; PC-MHI, Primary Care–Mental Health Integration program; PC-PTSD, Primary Care–Posttraumatic Stress Disorder Screen; PTSD, posttraumatic stress disorder.

^a^ Fisher exact tests were used when expected values in any cell of the contingency table were less than 5, and χ^2^ tests were used when all expected values in the contingency table were 5 or more.

^b^ Results from the Kruskal-Wallis test.

A total of 46 759 PC-PTSD screens were excluded from the analysis. Excluded screens were less likely to have had positive results compared with included screens (9.1% vs 13.8%, respectively; χ^2^ *P* < .001). Among those excluded, 42 948 screens were able to be linked to a patient’s vital status. An analysis of those patients indicated that they were more likely to die by suicide through December 31, 2016, compared with patients whose screens were included (0.3% vs 0.1%, respectively; *P* < .001).

Among 166 685 patients with positive initial PC-PTSD results in 2014, 88.5% were male, 64.7% were White, and 34.8% were 55 years or older. Among 1 385 896 patients with negative initial PC-PTSD results, 91.5% were male, 75.1% were White, and 65.9% were 55 years or older. In the bivariate analysis, among patients with positive initial PC-PTSD results, 0.13% died by suicide compared with 0.09% of patients with negative initial PC-PTSD results (*P* < .001).

 In the analysis unadjusted for covariates, a positive PC-PTSD result was associated with a 90% increase in the risk of suicide mortality at 1 day after screening (hazard ratio [HR], 1.90; 95% CI, 1.44-2.52) compared with a negative result (model 1 in Table 2). This risk decreased throughout the follow-up period (β for interaction = −0.0006; *P* = .04), which was up to 1095 days. The unadjusted suicide-specific survival probabilities are shown in the Figure. In the analysis adjusted for all covariates, a positive PC-PTSD result was associated with a 58% increase in the risk of suicide mortality at 1 day after screening (HR, 1.58; 95% CI, 1.19-2.10), and this risk decreased over time (β for interaction = −0.0006; *P* = .03) (model 5 in Table 2). At 30 days after screening, the HR for a positive PC-PTSD result was 1.55 (95% CI, 1.18- 2.04). At 365 days after screening, the HR was 1.26 (95% CI, 1.07-1.48), which represented a 26% increase. The HR at 475 days after screening was nonsignificant (HR, 1.17; 95% CI, 0.99-1.38). Covariate results are available in eTable 2 in the Supplement.

**Table 2.** Proportional Hazards Regression Analysis by Positive vs Negative Results on the Primary Care–Posttraumatic Stress Disorder Screens^a^

Model	Positive PC-PTSD, baseline HR (95% CI)	Interaction between positive PC-PTSD and time		HR over time (95% CI)			
		β (SE)	*P* value	Day 1	Day 30	Day 365	Day 500
1: Unadjusted	1.91 (1.44-2.52)	−0.0006 (0.0003)	.04	1.90 (1.44-2.52)	1.87 (1.43-2.44)	1.53 (1.31-1.78)	1.41 (1.21-1.64)
2: Adjusted for age and sex	1.90 (1.43-2.53)	−0.0006 (0.0003)	.03	1.90 (1.43-2.53)	1.87 (1.43-2.45)	1.52 (1.30-1.77)	1.40 (1.19-1.64)
3: Adjusted for all demographic characteristics	2.10 (1.57-2.80)	−0.0006 (0.0003)	.03	2.10 (1.57-2.79)	2.06 (1.57-2.71)	1.67 (1.42-1.96)	1.54 (1.30-1.81)
4: Adjusted for demographic characteristics and mental health diagnoses	1.63 (1.22-2.17)	−0.0006 (0.0003)	.03	1.63 (1.22-2.17)	1.60 (1.22-2.10)	1.30 (1.11-1.53)	1.19 (1.01-1.41)
5: Adjusted for demographic characteristics, mental health diagnoses, treatment, and suicide attempts	1.58 (1.19-2.11)	−0.0006 (0.0003)	.03	1.58 (1.19-2.10)	1.55 (1.18-2.04)	1.26 (1.07-1.48)	1.16 (0.98-1.36)

Abbreviation: HR, hazard ratio; PC-PTSD, Primary Care–Posttraumatic Stress Disorder Screen.

^a^ Includes 1 693 449 total screens.

**Figure.**
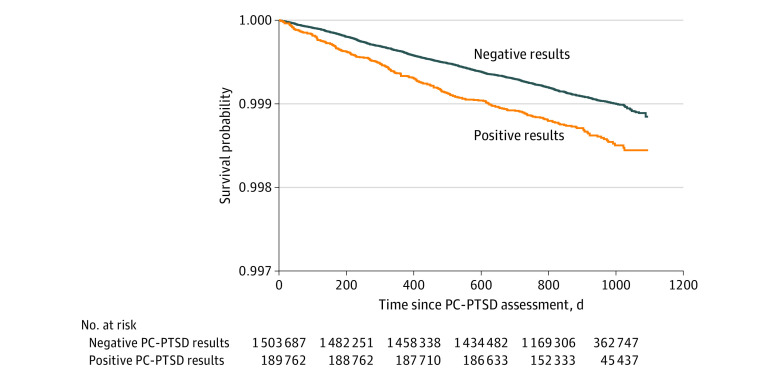
Kaplan-Meier Estimates of Survival by Positive vs Negative Results on the Primary Care–Posttraumatic Stress Disorder Screen (PC-PTSD)

In the unadjusted analysis examining each PC-PTSD item separately, a positive (yes) response to item 4 (“felt numb or detached from others, activities, or your surroundings”) was associated with a 117% increase in the risk of suicide mortality at 1 day after screening (HR, 2.17; 95% CI, 1.61-2.92), and this risk decreased over time (β for interaction = −0.0007; *P* = .009) (model 1 in Table 3). In the analysis adjusted for all covariates, a positive response to item 4 was associated with a 70% increase in the risk of suicide mortality at 1 day after screening (HR, 1.70; 95% CI, 1.27-2.28), and this risk also decreased over time (β for interaction = −0.0007; *P* = .007) (model 5 in Table 3). At 30 days after administration of the PC-PTSD, the HR for suicide mortality associated with a positive response to item 4 was 1.67 (95% CI, 1.26-2.21). At 365 days after screening, the HR was 1.31 (95% CI, 1.07-1.59), and the HR at 460 days after screening was nonsignificant (HR, 1.22; 95% CI, 0.99-1.48). Covariate results are available in eTable 3 in the Supplement. A post hoc test for trend revealed a 14% increase in suicide mortality risk for each unit increase in the PC-PTSD score (baseline HR, 1.14; 95% CI, 1.05-1.23; β for interaction = −0.0002; *P* = .01) (model 5 in Table 4).

**Table 3.** Proportional Hazards Regression Analysis by Positive Response for Each Item on the Primary Care–Posttraumatic Stress Disorder Screen^a^

Model	Positive PC-PTSD response, baseline HR (95% CI)				Interaction between item 4 and time^b^		HR over time for item 4 (95% CI)				
	Item 1	Item 2	Item 3	Item 4	β (SE)	*P* value	Day 1	Day 30	Day 365	Day 500
1: Unadjusted	0.95 (0.76-1.17)	1.14 (0.91-1.44)	0.91 (0.75-1.10)	2.17 (1.61-2.93)	−0.0007 (0.0003)	.009	2.17 (1.61-2.92)	2.12 (1.59-2.83)	1.67 (1.36-2.06)	1.52 (1.23-1.88)
2: Adjusted for age and sex	0.94 (0.76-1.17)	1.17 (0.93-1.48)	0.89 (0.73-1.09)	2.19 (1.62-2.96)	−0.0007 (0.0003)	.008	2.18 (1.62-2.96)	2.14 (1.60-2.86)	1.68 (1.36-2.07)	1.52 (1.23-1.89)
3: Adjusted for all demographic characteristics	0.99 (0.80-1.22)	1.22 (0.97-1.54)	0.93 (0.76-1.13)	2.15 (1.59-2.90)	−0.0007 (0.0003)	.007	2.15 (1.59-2.90)	2.10 (1.58-2.81)	1.65 (1.34-2.03)	1.50 (1.21-1.85)
4: Adjusted for demographic characteristics and mental health diagnoses	0.93 (0.76-1.14)	1.17 (0.94-1.46)	0.90 (0.74-1.08)	1.76 (1.31-2.36)	−0.0007 (0.0003)	.007	1.76 (1.31-2.36)	1.72 (1.30-2.28)	1.35 (1.11-1.64)	1.22 (1.00-1.50)
5: Adjusted for demographic characteristics, mental health diagnoses, treatment, and suicide attempts	0.92 (0.75-1.12)	1.17 (0.94-1.45)	0.90 (0.75-1.08)	1.70 (1.27-2.29)	−0.0007 (0.0003)	.007	1.70 (1.27-2.28)	1.67 (1.26-2.21)	1.31 (1.07-1.59)	1.18 (0.97-1.45)

Abbreviations: HR, hazard ratio; PC-PTSD, Primary Care–Posttraumatic Stress Disorder Screen.

^a^ Includes 1 693 449 total screens.

^b^ Interaction between item 4 and time was the only interaction term included in the final models because none of the other items had significant results in any of the models performed.

**Table 4.** Proportional Hazards Regression Analysis of Trends by Score on the Primary Care–Posttraumatic Stress Disorder Screen^a^

Model	PC-PTSD score, baseline HR (95% CI)	Interaction between PC-PTSD score and time		HR at day 1 (95% CI)			
		β (SE)	P value	Score of 1	Score of 2	Score of 3	Score of 4
1: Unadjusted	1.21 (1.12-1.31)	−0.0002 (0.0001)	.01	1.21 (1.12-1.30)	1.46 (1.26-1.70)	1.77 (1.41-2.22)	2.14 (1.58-2.90)
2: Adjusted for age and sex	1.21 (1.12-1.31)	−0.0002 (0.0001)	.01	1.21 (1.12-1.31)	1.47 (1.26-1.71)	1.78 (1.42-2.24)	2.16 (1.59-2.94)
3: Adjusted for all demographic characteristics	1.25 (1.16-1.35)	−0.0002 (0.0001)	.01	1.25 (1.16-1.35)	1.56 (1.34-1.82)	1.95 (1.54-2.46)	2.44 (1.78-3.33)
4: Adjusted for demographic characteristics and mental health diagnoses	1.15 (1.06-1.25)	−0.0002 (0.0001)	.01	1.15 (1.06-1.25)	1.32 (1.13-1.55)	1.53 (1.20-1.94)	1.76 (1.28-2.41)
5: Adjusted for demographic characteristics, mental health diagnoses, treatment, and suicide attempts	1.14 (1.05-1.23)	−0.0002 (0.0001)	.01	1.14 (1.05-1.23)	1.30 (1.10-1.52)	1.47 (1.16-1.87)	1.68 (1.22-2.31)

Abbreviations: HR, hazard ratio; PC-PTSD, Primary Care–Posttraumatic Stress Disorder Screen.

^a^ Includes 1 693 449 total screens.

## Discussion

Among patients who received PC-PTSD screens through the VHA health system in 2014, positive PC-PTSD results were associated with an increased risk of suicide mortality through 1 year after screening. We observed a significant dose-response pattern in the association between PC-PTSD score and suicide mortality, with greater risk observed as the number of positive responses to PC-PTSD items increased. We found a positive association between PTSD symptoms and suicide mortality even in analyses that were controlled for several mental health diagnoses. This pattern differed from that of previous studies examining PTSD diagnoses and suicide mortality.^16,20,21,22,23^ Our findings indicate that veterans who receive positive results on the PC-PTSD have an increased risk of suicide mortality. These findings may inform treatment decisions and support the need for continued improvement of suicide risk assessment.

We note that our sample consisted of patients who did not have a formal diagnosis of PTSD yet remained at an increased risk of suicide mortality. A PTSD diagnosis requires the presence of symptoms across 4 domains: reexperiencing, avoidance, negative alterations in cognition and mood, and hyperarousal. These symptoms must persist for more than 1 month.^41^ However, the presence of subthreshold PTSD symptoms may indicate suicide risk regardless of whether a patient qualifies for a formal diagnosis.^42^ In the absence of a PTSD diagnosis, a positive PC-PTSD result may indicate subthreshold symptoms that suggest a patient has an increased risk of suicide mortality. Patients with a positive PC-PTSD result may also have met the full diagnostic criteria for PTSD, which had not been detected until the time of screening.^43^ As a consequence, in lieu of waiting for a formal diagnosis, it may be important to consider initiating suicide risk assessment strategies for patients with positive responses on the PC-PTSD. Another reason to use and incorporate the PC-PTSD into suicide risk assessment strategies is that, unlike a medical records diagnosis, the PC-PTSD asks a patient about PTSD symptoms experienced within the past month. The symptom severity of PTSD may fluctuate daily,^25^ and this fluctuation has been linked to adverse health outcomes.^44^ Although further research is warranted, findings from the present analysis suggest that the PC-PTSD might be a better indicator of immediate suicide mortality risk than a static diagnosis obtained from the medical record. Future research is needed to examine differences in suicide mortality risk among those who receive a formal PTSD diagnosis after administration of the PC-PTSD.

Additional risk was associated with a positive response to item 4 (“felt numb or detached from others, activities, or your surroundings”). This symptom has been associated with suicidal ideation in previous research.^26,28,30,45,46^ A positive response to item 4 may indicate feelings of social isolation or thwarted belongingness, which may be one of the most important factors in suicidal desire, as described by the interpersonal theory of suicide.^47,48^ This finding highlights the need to improve social support systems and encourage engagement in meaningful activities, which may not only reduce PTSD symptoms but potentially help to mitigate the risk of suicide.

In 2018, the VHA implemented a new strategy for risk assessment that included the addition of 1 measure to the PC-PTSD. This measure, item 9 from the Patient Health Questionnaire, asks patients about suicidal ideation. Positive responses to that item have been associated with an increase in suicide risk.^49,50^ The VHA requires additional risk evaluation for patients with positive responses to item 9. The findings of the present study raise the possibility that positive PC-PTSD results may also be associated with greater suicide mortality risk. As more current mortality data become available, future analyses will assess risk using the expanded instrument to evaluate this issue and inform ongoing VHA health system suicide prevention activities.

### Limitations

This study has several study limitations. We examined PC-PTSD data available in VHA medical records, and these data may not be generalizable to other populations. Although the sample included all screens coded in the administrative data for 2014, we may not have captured every PC-PTSD administered within the VHA health care system in 2014. We noted in the Results section that patients with excluded screens had an increased risk of suicide mortality. Excluding their screens may have inflated the study results, as excluded screens were less likely to have had positive overall results compared with included screens. In addition, we did not assess depressive symptoms, which may be associated with suicide mortality risk, at the time of PC-PTSD administration. The PC-PTSD has been updated based on the *Diagnostic and Statistical Manual of Mental Disorders, Fifth Edition*^51^; this update, the PC-PTSD-5, has been used in the VHA health care system since 2018. Future research is needed to examine the associations between PC-PTSD-5 results and suicide among patients receiving care in the VHA system.

## Conclusions

To our knowledge, this is the first study to examine the association between positive PC-PTSD results and suicide mortality. Positive PC-PTSD results, and specifically positive responses on items that describe feeling numb or detached, were associated with an increase in the risk of suicide mortality, which decreased over time. The PC-PTSD may identify distinct risks that are not identified by other assessment procedures used in the VHA or other health systems.

## Notes

Supplement. **eTable 1.** *ICD-9-CM* Codes for Mental Health Diagnoses **eTable 2.** Covariate Results of Proportional Hazards Regression Models Regarding Risk of Suicide Death for 2014 VHA PC-PTSD Screens—Positive vs Negative PC-PTSD Screens **eTable 3.** Covariate Results of Proportional Hazards Regression Models Regarding Risk of Suicide Death for 2014 VHA PC-PTSD Screens—PC-PTSD Individual Item Responses
