# Error in Methods

**DOI:** 10.1001/jamanetworkopen.2020.26185

**Published:** 2020-10-15

In the Original Investigation titled “Association Between Positive Results on the Primary Care–Posttraumatic Stress Disorder Screen and Suicide Mortality Among US Veterans,”^1^ published September 3, 2020, there was an error in the study approval portion of the Methods section. The text “with a waiver of informed consent” was added by mistake. The study did not require approval or consent because it was a quality improvement project. This article has been corrected.^1^
