# Supplementary material for: Association Between Positive Results on the Primary Care–Posttraumatic Stress Disorder Screen and Suicide Mortality Among US Veterans
Source: JAMA Netw Open. 2020 Sep 3;3(9):e2015707. doi: 10.1001/jamanetworkopen.2020.15707 (PMC7489804; doi:10.1001/jamanetworkopen.2020.15707)
Supplement: Supplement. — eTable 1. ICD-9-CM Codes for Mental Health Diagnoses eTable 2. Covariate Results of Proportional Hazards Regression Models Regarding Risk of Suicide Death for 2014 VHA PC-PTSD Screens—Positive vs Negative PC-PTSD Screens eTable 3. Covariate Results of Proportional Hazards Regression Models Regarding Risk of Suicide Death for 2014 VHA PC-PTSD Screens—PC-PTSD Individual Item Responses [file jamanetwopen-e2015707-s001.pdf]

Supplementary Online Content

Cooper SA, Szymanski BR, Bohnert KM, Sripada RK, McCarthy JF. Association between positive results on the Primary Care–Posttraumatic Stress Disorder Screen and suicide mortality among US veterans. *JAMA Netw Open*. 2020;3(9):e2015707. doi:10.1001/jamanetworkopen.2020.15707

eTable 1. *ICD-9-CM* Codes for Mental Health Diagnoses

eTable 2. Covariate Results of Proportional Hazards Regression Models Regarding Risk of Suicide Death for 2014 VHA PC-PTSD Screens—Positive vs Negative PC-PTSD Screens

eTable 3. Covariate Results of Proportional Hazards Regression Models Regarding Risk of Suicide Death for 2014 VHA PC-PTSD Screens—PC-PTSD Individual Item Responses

This supplementary material has been provided by the authors to give readers additional information about their work.

© 2020 Cooper SA et al. *JAMA Network Open*.

eTable 1. *ICD-9-CM* Codes for Mental Health Diagnoses

**Mental Health Diagnosis**	***ICD-9-CM* Codes**
Posttraumatic Stress Disorder	309.81
Substance Use Disorder	291.x, 292.x, 303.0x, 303.9x, 304.0x, 304.1x, 304.2x, 304.3x, 304.4x, 304.5x, 304.6x, 304.7x, 304.8x, 304.9x, 305.0x, 305.2x, 305.3x, 305.4x, 305.5x, 305.6x, 305.7x, 305.8x, 305.9x
Anxiety	293.84, 300.00, 300.01, 300.02, 300.09, 300.10, 300.20, 300.21, 300.22, 300.23, 300.29, 300.3x, 300.7x
Bipolar Disorder	296.0x, 296.1x, 296.4x, 296.5x, 296.6x, 296.7x, 296.8x
Depression	293.83, 296.2x, 296.3x, 296.90, 296.99, 298.0x, 300.4x, 301.12, 309.0x, 309.1x, 311.x
Schizophrenia	295.0x, 295.1x, 295.2x, 295.3x, 295.4x, 295.6x, 295.7x, 295.8x, 295.9x
Other Psychiatric Diagnosis	290.8-291.1, 291.3-292.81, 292.83-294.0, 294.9-305.0, 305.2-319 (excluding above categories)

© 2020 Cooper SA et al. *JAMA Network Open*.

eTable 2. Covariate Results of Proportional Hazards Regression Models Regarding Risk of Suicide Death for 2014 VHA PC-PTSD Screens—Positive vs Negative PC-PTSD Screens (N = 1,693,449)

	**Model 2: Adjusting for age and sex**		**Model 3: Adjusting for all demographics**		**Model 4: Adjusting for demographics and mental health diagnoses**		**Model 5: Adjusting for demographics, mental health diagnoses, treatment and suicide attempt**	
**Covariate**	Hazard Ratio	95% Confidence Interval	Hazard Ratio	95% Confidence Interval	Hazard Ratio	95% Confidence Interval	Hazard Ratio	95% Confidence Interval
Service-connected for post-traumatic stress disorder			0.72	0.56-0.93	0.84	0.66-1.07	0.86	0.67-1.10
Sex								
Male	3.11	2.35-4.12	3.02	2.28-4.00	3.22	2.43-4.28	3.27	2.46-4.34
Female	(ref)		(ref)		(ref)		(ref)	
Age Group								
18-34	(ref)		(ref)		(ref)		(ref)	
35-54	0.86	0.73-1.02	1.00	0.83-1.19	0.90	0.75-1.08	0.89	0.75-1.07
55-74	0.69	0.59-0.80	0.76	0.64-0.91	0.77	0.64-0.92	0.77	0.64-0.92
75+	1.05	0.88-1.26	1.20	0.97-1.49	1.41	1.14-1.74	1.43	1.16-1.78
Marital Status								
Divorced			(ref)		(ref)		(ref)	
Married			0.55	0.48-0.63	0.64	0.56-0.74	0.65	0.56-0.75
Never Married/Single			0.99	0.83-1.19	1.01	0.85-1.20	1.01	0.85-1.21
Separated			1.07	0.80-1.44	1.02	0.76-1.37	1.01	0.75-1.35
Widowed			0.79	0.60-1.04	0.86	0.65-1.14	0.87	0.66-1.15
Unknown			0.58	0.42-0.81	0.65	0.47-0.91	0.67	0.48-0.93
Race								

© 2020 Cooper SA et al. *JAMA Network Open*.

American Indian or Alaska Native			(ref)		(ref)		(ref)	
Asian			0.36	0.14-0.91	0.38	0.15-0.96	0.38	0.15-0.96
Black or African American			0.31	0.15-0.61	0.31	0.15-0.61	0.30	0.15-0.60
White			1.14	0.60-2.16	1.09	0.57-2.08	1.07	0.56-2.03
Two or More Races			1.42	0.73-2.77	1.01	0.52-1.98	0.89	0.46-1.75
Unknown			0.96	0.47-1.97	1.06	0.52-2.17	1.07	0.52-2.20
Mental Health Diagnoses								
Substance Use Disorder					1.59	1.36-1.87	1.38	1.16-1.64
Anxiety					1.43	1.22-1.67	1.22	1.02-1.45
Bipolar Disorder					2.54	1.99-3.24	1.75	1.31-2.34
Depression					1.82	1.58-2.11	1.51	1.28-1.77
Schizophrenia					1.65	1.18-2.30	1.11	0.78-1.59
Other Psychiatric Diagnosis					0.99	0.77-1.27	0.91	0.71-1.17
Encounters on Assessment Date								
Primary Care - Mental Health Integration (PC-MHI)							1.35	1.04-1.75
Non-PC-MHI Primary Care							1.02	0.83-1.25
Non-PC-MHI, Non-PC, Specialty Mental Health							1.33	1.07-1.65
Other Outpatient							0.97	0.87-1.08

© 2020 Cooper SA et al. *JAMA Network Open*.

Inpatient Mental Health							1.80	1.14-2.84
Other Inpatient							0.50	0.27-0.91
Inpatient MH Stay in the prior 365 days							0.91	0.61-1.34
ED Visit in the prior 365 days							1.11	0.93-1.33
Presence a Medication fill in the prior 365 days								
Antipsychotic							1.45	1.13-1.85
Antidepressant							1.09	0.92-1.28
Benzodiazepine							1.01	0.75-1.36
Mood Stabilizer							1.16	0.98-1.37
Anxiolytic/Sedative							1.31	1.00-1.72
Prior Suicide Attempt							3.08	1.91-.4.96
Number of Inpatient MH bed days in the prior 365 days							1.00	0.99-1.00
Number of ED visits in the prior 365 days							0.98	0.92-1.04

© 2020 Cooper SA et al. *JAMA Network Open*.

eTable 3. Covariate Results of Proportional Hazards Regression Models Regarding Risk of Suicide Death for 2014 VHA PC-PTSD Screens—PC-PTSD Individual Item Responses (N = 1,693,449)

	**Model 2: Adjusting for age and sex**		**Model 3: Adjusting for all demographics**		**Model 4: Adjusting for demographics and mental health diagnoses**		**Model 5: Adjusting for demographics, mental health diagnoses, treatment and suicide attempt**	
**Covariate**	Hazard Ratio	95% Confidence Interval	Hazard Ratio	95% Confidence Interval	Hazard Ratio	95% Confidence Interval	Hazard Ratio	95% Confidence Interval
Service-connected for post-traumatic stress disorder			0.71	0.56-0.92	0.85	0.66-1.08	0.87	0.68-1.12
Sex								
Male	3.12	2.36-4.13	3.02	2.28-4.00	3.23	2.43-4.29	3.28	2.47-4.35
Female	(ref)		(ref)		(ref)		(ref)	
Age Group								
18-34	(ref)		(ref)		(ref)		(ref)	
35-54	0.87	0.73-1.03	1.01	0.84-1.20	0.90	0.75-1.08	0.89	0.74-1.07
55-74	0.70	0.60-0.82	0.78	0.65-0.94	0.77	0.64-0.92	0.77	0.64-0.92
75+	1.09	0.91-1.31	1.24	1.00-1.54	1.41	1.14-1.75	1.43	1.15-1.78
Marital Status								
Divorced			(ref)		(ref)		(ref)	
Married			0.55	0.48-0.64	0.64	0.56-0.74	0.65	0.57-0.75
Never Married/Single			1.00	0.83-1.19	1.01	0.84-1.20	1.01	0.85-1.21
Separated			1.07	0.79-1.44	1.02	0.76-1.37	1.01	0.75-1.35
Widowed			0.79	0.60-1.05	0.86	0.66-1.14	0.87	0.66-1.15
Unknown			0.58	0.42-0.81	0.65	0.47-0.91	0.67	0.48-0.93
Race								

© 2020 Cooper SA et al. *JAMA Network Open*.

American Indian or Alaska Native			(ref)		(ref)		(ref)	
Asian			0.36	0.14-0.92	0.38	0.15-0.96	0.37	0.15-0.95
Black or African American			0.31	0.16-0.61	0.31	0.15-0.61	0.30	0.15-0.60
White			1.14	0.60-2.18	1.09	0.57-2.08	1.06	0.56-2.03
Two or More Races			1.42	0.73-2.77	1.01	0.52-1.98	0.89	0.46-1.74
Unknown			0.97	0.47-1.98	1.06	0.52-2.16	1.07	0.52-2.20
Mental Health Diagnoses								
Substance Use Disorder					1.59	1.35-1.86	1.38	1.16-1.64
Anxiety					1.43	1.22-1.67	1.22	1.02-1.45
Bipolar Disorder					2.53	1.98-3.22	1.74	1.30-2.33
Depression					1.81	1.56-2.09	1.50	1.28-1.76
Schizophrenia					1.65	1.18-2.30	1.12	0.78-1.59
Other Psychiatric Diagnosis					0.99	0.77-1.27	0.92	0.71-1.18
Encounters on Assessment Date								
Primary Care - Mental Health Integration (PC-MHI)							1.34	1.04-1.74
Non-PC-MHI Primary Care							1.02	0.83-1.25
Non-PC-MHI, Non-PC, Specialty Mental Health							1.32	1.07-1.64
Other Outpatient							0.97	0.88-1.08

© 2020 Cooper SA et al. *JAMA Network Open*.

Inpatient Mental Health							1.79	1.14-2.83
Other Inpatient							0.50	0.27-0.91
Inpatient MH Stay in the prior 365 days							0.91	0.61-1.35
ED Visit in the prior 365 days							1.11	0.93-1.33
Presence a Medication fill in the prior 365 days								
Antipsychotic							1.45	1.14-1.85
Antidepressant							1.09	0.92-1.28
Benzodiazepine							1.01	0.75-1.36
Mood Stabilizer							1.16	0.98-1.37
Anxiolytic/Sedative							1.32	1.01-1.72
Prior Suicide Attempt							3.07	1.90-4.94
Number of Inpatient MH bed days in the prior 365 days							1.00	0.99-1.00
Number of ED visits in the prior 365 days							0.98	0.92-1.04

© 2020 Cooper SA et al. *JAMA Network Open*.
